# Regulating the human HECT E3 ligases

**DOI:** 10.1007/s00018-018-2848-2

**Published:** 2018-06-01

**Authors:** Jasper Sluimer, Ben Distel

**Affiliations:** 1 Department of Medical Biochemistry, Academic Medical Center, University of Amsterdam, Amsterdam, The Netherlands; 2 Department of Neuroscience, Erasmus Medical Center, Wijtemaweg 80, 3015 CN Rotterdam, The Netherlands

**Keywords:** Ubiquitination, HECT E3 ligase, Substrate recruitment, Intramolecular interaction, Activity regulation, Oligomerization, Post-translational modification

## Abstract

Ubiquitination, the covalent attachment of ubiquitin to proteins, by E3 ligases of the HECT (homologous to E6AP C terminus) family is critical in controlling diverse physiological pathways. Stringent control of HECT E3 ligase activity and substrate specificity is essential for cellular health, whereas deregulation of HECT E3s plays a prominent role in disease. The cell employs a wide variety of regulatory mechanisms to control HECT E3 activity and substrate specificity. Here, we summarize the current understanding of these regulatory mechanisms that control HECT E3 function. Substrate specificity is generally determined by interactions of adaptor proteins with domains in the N-terminal extensions of HECT E3 ligases. These N-terminal domains have also been found to interact with the HECT domain, resulting in the formation of inhibitory conformations. In addition, catalytic activity of the HECT domain is commonly regulated at the level of E2 recruitment and through HECT E3 oligomerization. The previously mentioned regulatory mechanisms can be controlled through protein–protein interactions, post-translational modifications, the binding of calcium ions, and more. Functional activity is determined not only by substrate recruitment and catalytic activity, but also by the type of ubiquitin polymers catalyzed to the substrate. While this is often determined by the specific HECT member, recent studies demonstrate that HECT E3s can be modulated to alter the type of ubiquitin polymers they catalyze. Insight into these diverse regulatory mechanisms that control HECT E3 activity may open up new avenues for therapeutic strategies aimed at inhibition or enhancement of HECT E3 function in disease-related pathways.

## Introduction

**Fig. 1:**
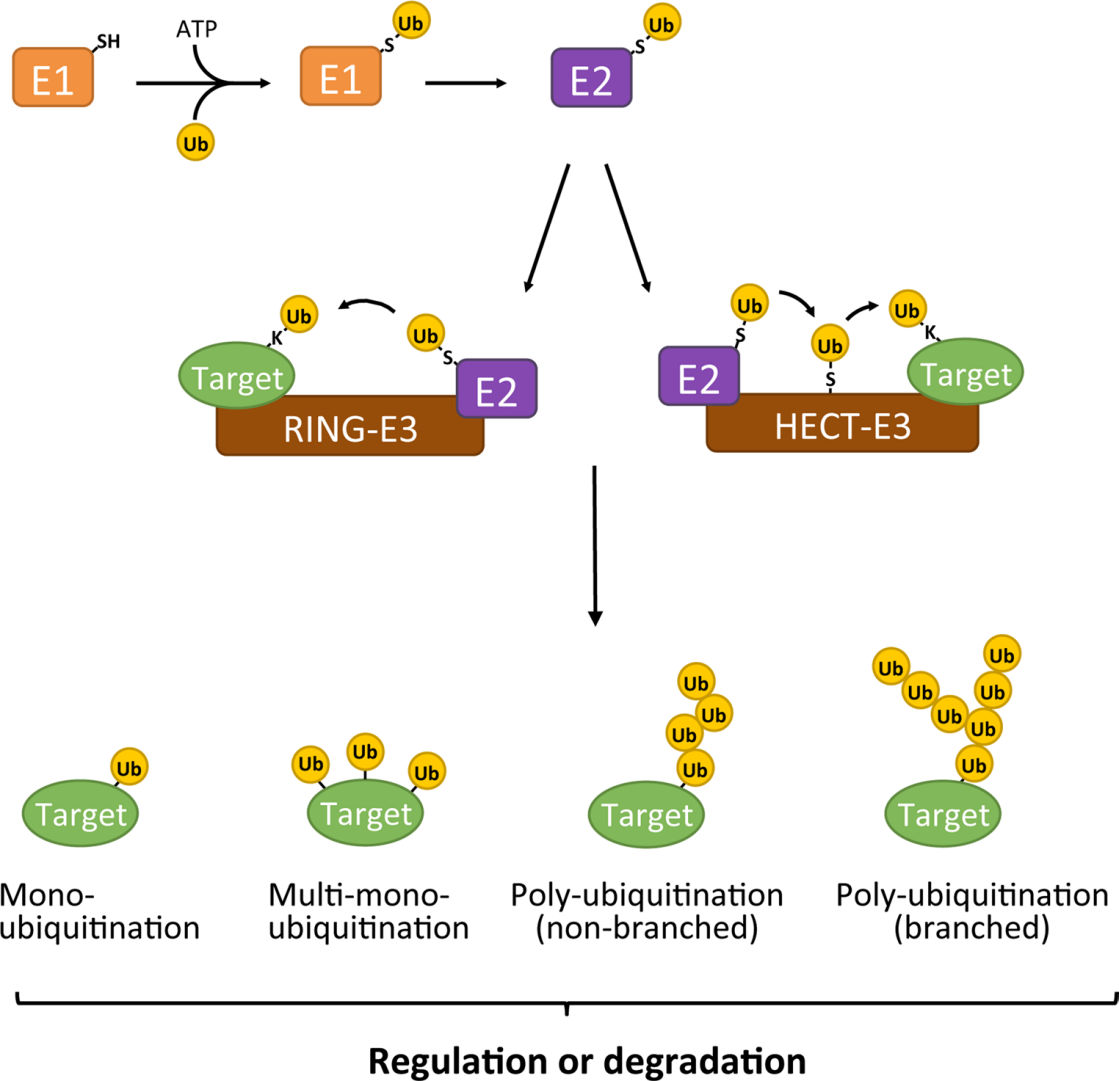
The ubiquitination cascade. The initial step involves the ATP-dependent transfer of ubiquitin to an active-site cysteine residue on the E1 ubiquitin-activating enzyme. In the next step, the ubiquitin is transferred from the E1 to the E2 ubiquitin-conjugating enzyme. Once the E2 is charged with ubiquitin, it can associate with the E3 to prepare the ubiquitin transfer to the target protein. A RING-E3 will mediate a direct transfer of ubiquitin from the E2 to the target protein. The substrate ubiquitination by HECT E3s (and RBR ligases, not shown) involves an additional step where the ubiquitin is first chemically bound to an active-site cysteine of the HECT domain before it is attached to the target protein. Target proteins can be modified by mono-, multi-mono-, or polyubiquitin. As ubiquitin has seven internal lysine residues and an N-terminal amino group, all of which can be ubiquitinated, a wide variety of polyubiquitin chains can be formed (not shown). Depending on the type of (poly)ubiquitin modification, either the function/localization of the target protein is changed (“regulation”) or the target protein is sent for degradation by the 26S proteasome (“degradation”)

Ubiquitination is a post-translational modification that is important for regulating protein function and degradation. The ubiquitination cascade comprises the sequential actions of the ubiquitin-activating enzyme (E1), ubiquitin-conjugating enzymes (E2s), and ubiquitin ligases (E3s) (Fig. 1). Within the ubiquitin cascade, the E3 ligases primarily determine specificity regarding selection of target proteins and ubiquitination sites. E3s are often focal points of cellular regulation and this makes them attractive targets for therapeutic intervention [1–3].

E3 ligases are commonly grouped into three classes: really interesting new genes (RINGs), homologous to E6AP C terminus (HECTs), and RING-between-RINGs (RBRs). While the three classes of E3 ligases all catalyze covalent attachment of ubiquitin to usually a Lys residue in the target protein, they differ in structure and mechanisms (reviewed in [4]). A notable distinction in the mechanism between the classes is that RING E3s catalyze a direct transfer of ubiquitin from the E2 to the target protein, whereas transfer of ubiquitin by HECT E3s (and RBR ligases) involves and intermediate step where the ubiquitin is first transferred from the E2 to an active-site cysteine residue on the HECT E3 ligase before it is conjugated to the target protein. Target proteins can be modified by a single ubiquitin moiety on one or multiple sites, giving rise to mono- and multi-mono-ubiquitinated proteins, respectively. In addition, a wide variety of polyubiquitin chains can be formed on target proteins, in which the ubiquitin moieties can be linked through either one of the seven internal Lys residues (Lys6, Lys11, Lys27, Lys29, Lys33, Lys48, or Lys63) in ubiquitin or through its N-terminal amino group. The consequences for the modified target protein are determined by the type of (poly)ubiquitin modification it received. Ubiquitination can result in the change of function, localization or activity of the modified protein, or control its degradation via the 26S proteasome (reviewed in [5]).

The identification of the E6AP protein transcribed from the ubiquitin-protein ligase E3A (UBE3A) gene led to the discovery of the HECT-type E3 ligase family [6–8]. HECT E3s are directly implicated in cancer, hypertension, neurodegenerative disorders, and other diseases, such as Angelman syndrome which is caused by the loss of maternally inherited UBE3A [9, 10]. E6AP was discovered through its interaction with the human papillomavirus protein (HPV) E6, which hijacks the E3 ligase to ubiquitinate the p53 tumor suppressor as well as several other cellular proteins resulting in their degradation [11, 12]. In a non-infected cell p53 is not a target of E6AP, but the E6 protein alters the substrate specificity of the E3 ligase to target and ubiquitinate p53 [7]. The interaction between the E6 protein and E6AP is one example of how a HECT E3 ligase is activated and altered to ubiquitinate a specific substrate through binding of an adaptor protein.

**Fig. 2:**
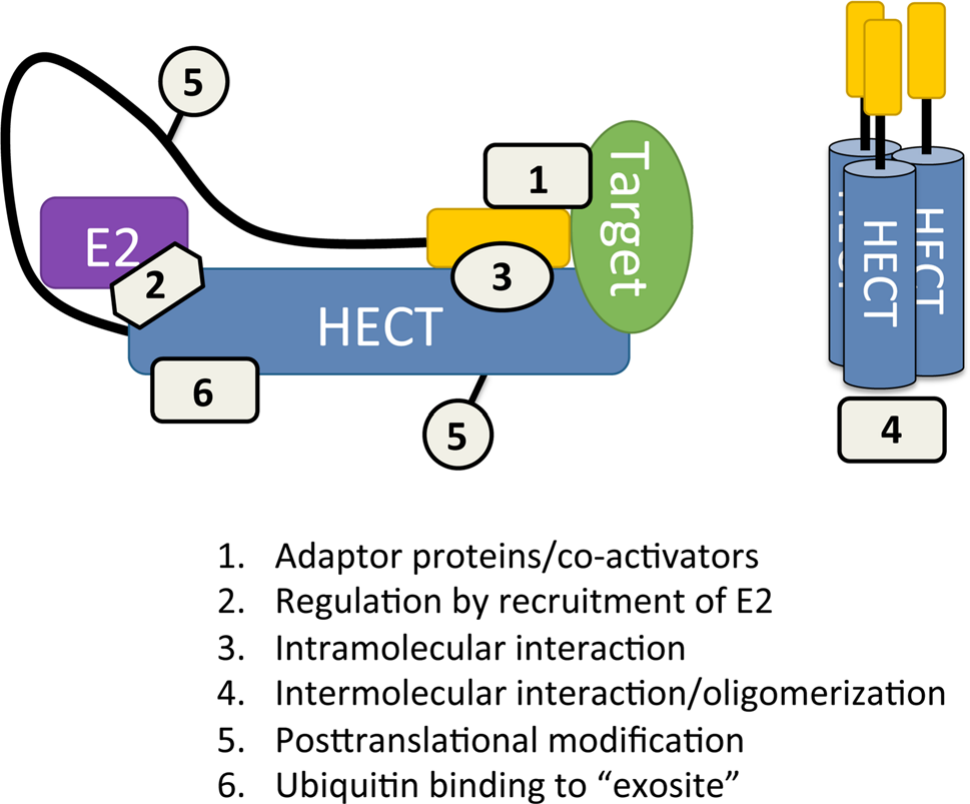
Modes of regulating HECT E3 ligase function. Mechanisms that can regulate or modulate HECT E3 ligase function are: (1) recruitment of substrate and activity modulation by adaptor proteins/co-activators, (2) recruitment of E2, (3) intramolecular interaction between an N-terminal domain and the HECT domain, (4) intermolecular interaction/oligomerization, (5) post-translational modification, and (6) ubiquitin binding to the “exosite” on the HECT domain. For details see text

The interaction with adaptor proteins, such as the interaction of E6 with E6AP, can not only change the substrate specificity of HECT E3s but also alter the structure of the ubiquitin polymers they conjugate to the substrates. Post-translational modifications (PTMs) are also prevalent in the alteration of HECT E3 ligase functionality such as phosphorylation and the attachment of ubiquitin-like modifiers (UBLs) [13–15]. Various mechanisms other than PTMs and interactions with adaptor proteins also regulate HECT E3 ligases such as intramolecular interactions, oligomerization, and recruitment of the E2 (Fig. 2).

This review aims to give a comprehensive overview of the different mechanisms through which HECT E3 ligase activities and substrate specificities are regulated. The structural aspects of these regulatory mechanisms have been very recently surveyed [16], while the pathophysiological aspects of HECT E3 ligases have been addressed by Scheffner and Kumar [17]. In addition to providing a clearer perspective of the regulation of HECT E3 ligases, this overview could show us which aspects of research on HECT E3s have made good progress and which areas of research have lagged behind. Together, these insights may lead to suggestions for future research and pave the way for new therapeutic strategies for many diseases.

## HECT E3 ligases

HECT E3 ligases can be distinguished from other classes of ubiquitin E3 ligases in that they have an active-site cysteine that forms an intermediate thioester bond with ubiquitin before the ubiquitin is linked to its substrate [8, 18]. RING E3s do not have this catalytic activity, but rather act as allosteric activators of E2s that mediate the transfer of ubiquitin from E2 to substrate directly [19]. This direct transfer of ubiquitin from the E2 means that the linkage type of the ubiquitin chains catalyzed by RING E3s is generally determined by the specific E2 conjugating enzyme [20]. In contrast, HECT E3 ligases form an E3 ~ Ub intermediate prior to the transfer of ubiquitin to the substrate, allowing them to override any linkage-type preferences that an E2 conjugating enzyme may have [21]. Consistent with this, some HECT E3s appear to have a general preference for catalyzing chains of specific linkage types even when they cooperate with different E2s: E6AP predominantly assembles Lys48-linked chains [21–23]; Rsp5 and NEDD4.1 assemble preferentially Lys63-linked chains [21–23]; UBE3C promotes formation of Lys29- and Lys48-linked chains [22]; and recently it was shown that WWP1 assembles ubiquitin chains containing Lys63, Lys48, and Lys11 linkages [24]. For most other HECT E3s their linkage-type preferences, if any, remain to be discovered. Along with ubiquitin, HECT E3s are found in all eukaryotic organisms. HECT domain-like E3 proteins have also been found in pathogenic bacteria [25]. These bacteria presumably exploit the ubiquitin system of their host cells by injecting them with the respective HECT domain-like E3s [26]. Another characteristic of some HECT E3 ligases is that they are capable of catalyzing UBL proteins to their substrates such as NEDD8 (neural precursor cell expressed developmentally down-regulated protein 8) [14] and ISG15 (ubiquitin-like modifier IFN-stimulated gene 15) [27–29].

## The HECT domain

**Fig. 3:**
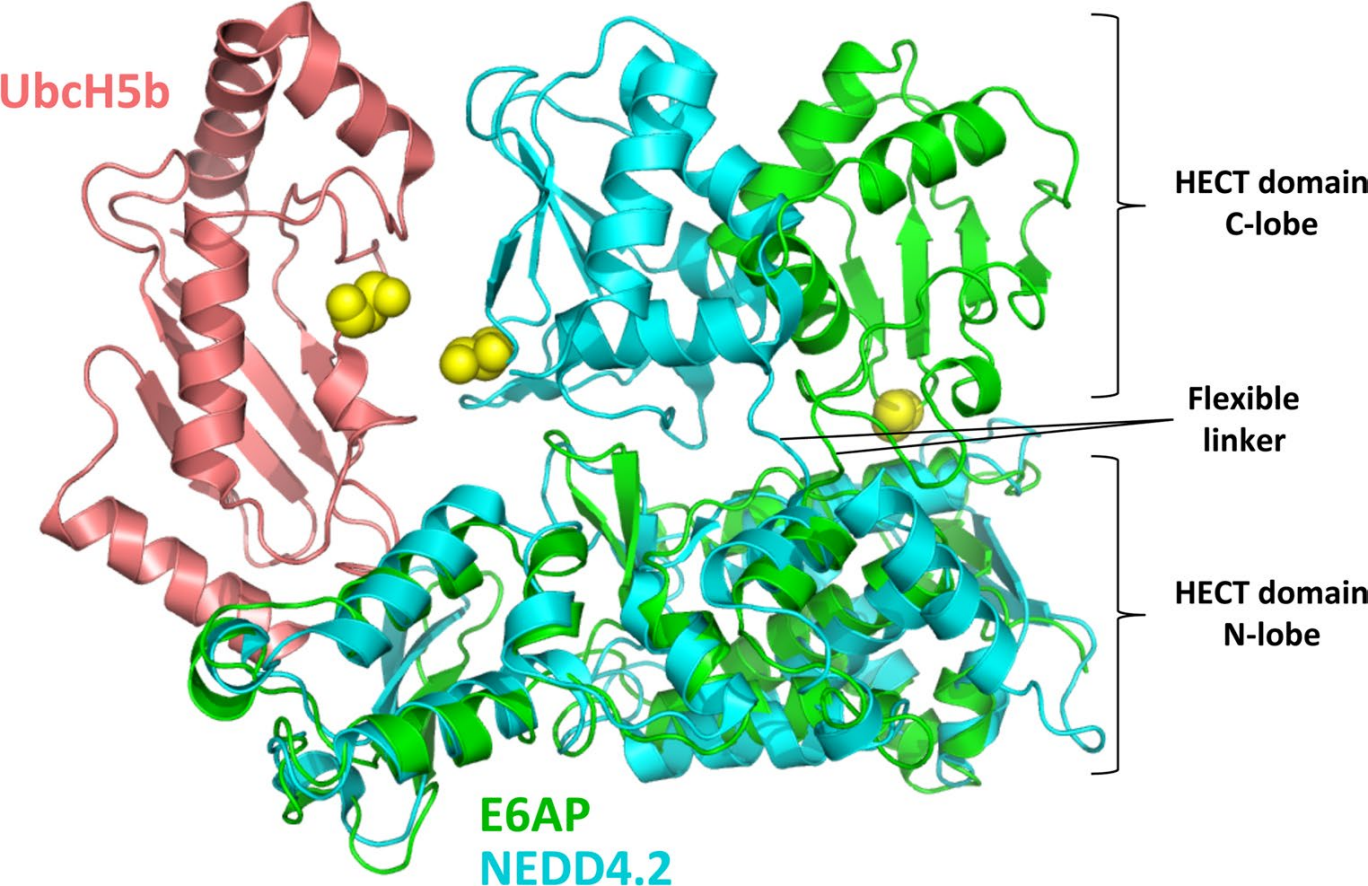
Structural rearrangements in the catalytic HECT domain. Illustration of alternate HECT domain C-lobe positions as seen in the crystal structures of UbcH7 (not shown for clarity)-E6AP^HECT^ (green) (PDB ID: 1C4Z) and UbcH5b (salmon) ~ Ubiquitin (not shown for clarity)-NEDD4.2^HECT^ (cyan)(PBD ID: 3JW0). The flexible linker that allows for rearrangements of the N- and C-lobes with respect to each other is indicated. Catalytic cysteine residues are displayed as yellow balls. For details see text

The HECT domain is an approximately 40 kDa domain positioned at the C-terminal of the E3 ligases that consists of two flexibly tethered lobes (the N- and C-lobes). Across the HECT family, there is a 16–92% amino-acid identity for the domain [30]. The larger N-lobe (approximately 250 amino acids) contains the docking surface for the E2 [31]. The short flexible hinge connects the N-lobe to the shorter C-lobe, which contains the active-site cysteine. Non-covalent interactions between the E2 and the N- and C-lobes influence the conformation of the HECT-E2 complex depending on the ubiquitin-loading status of the E2. A crystal structure of the E6AP HECT domain with an unloaded E2 shows a large distance between the E2 and HECT domain cysteines [31]. A subsequent structure of the NEDD4.2 HECT with an ubiquitin-loaded E2 revealed a large change in E2–E3 topology, bringing the cysteines of both proteins in close proximity [32] (Fig. 3). These structural rearrangements, which are required for catalysis, are dependent on the flexibility of the linker that connects the N- and C-lobes of the HECT domain [33]. Properties of the E2 docking surface on the HECT domain in combination with the E2-conjugating enzyme involved may determine the efficiency at which ubiquitin chains are elongated. This is presumed because polyubiquitin chain synthesis requires multiple E2–E3 binding events due to an overlapping binding domain on the E2 for E1 and E3 [34].

## HECT E3 families

**Fig. 4:**
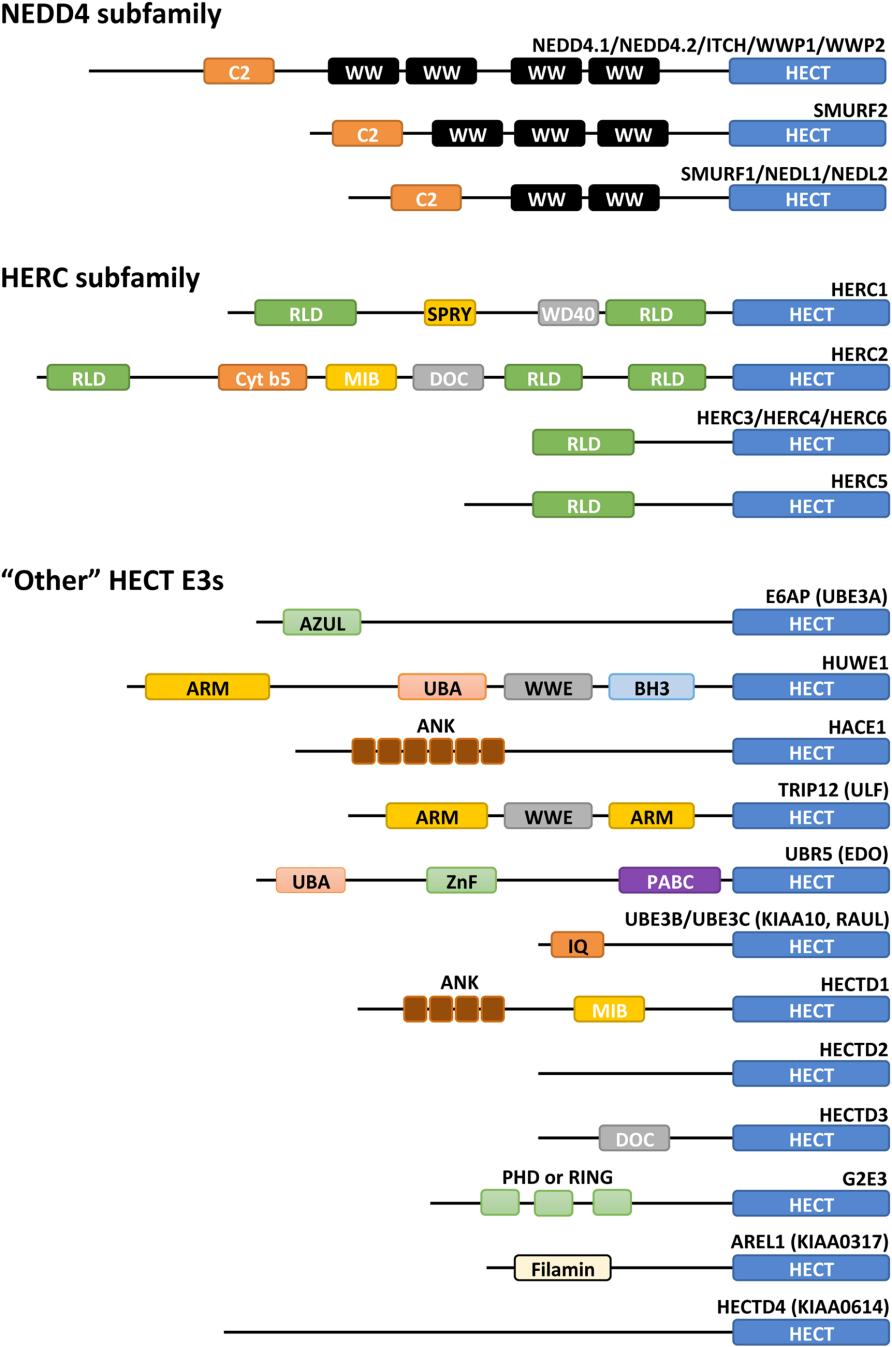
Human HECT E3 protein domain architecture. Overview of the domain organization of human HECT E3 ligases. Protein domains were predicted by the InterPro server [156]. HECT E3 ligases are characterized by the presence of a conserved HECT (homologous to E6AP C-terminus) domain that is located at the C-terminus of the proteins. The human HECT E3 ligase family is grouped into two subfamilies and 13 “other” HECT E3 ligases based on their domain architecture N-terminal to the HECT domain. The NEDD4 subfamily comprises nine members each having an N-terminal C2 domain and between two and four WW domains. The HERC subfamily has six members that each contains between one and three RLDs (RCC-like domains) while the two large family members, HERC1 and HERC2, additionally contain various other domains. The “other” HECT E3 ligases contain various domains as shown. Domain abbreviations used are as follows: *C2* C2 domain (Ca^2+^-binding domain), *WW* WW domain, *RLD* RCC-like domain, *SPRY* B30.2/SPRY domain, *WD40* W-D repeat domain, *Cytb5* cytochrome b5-like heme/steroid-binding domain, *DOC* APC10/DOC domain, *MIB* MIB-HERC2 domain, *AZUL* amino-terminal Zn-binding domain of UBE3A ligase, *ARM* Armadillo repeat-containing domain, *UBA* ubiquitin-associated domain, *WWE* WWE domain, *BH3* Bcl-2 homology 3 domain, *ANK* Ankyrin repeat-containing domain, *ZnF* Zinc finger domain, *PABC* polyadenylate-binding protein C-terminal domain, *IQ* IQ motif/EF-hand binding site, *PHD* PHD-type zinc finger, *Filamin* filamin/ABP280 repeat-like domain

Among the various HECT E3 ligases, a large variety of configurations are observed in the region located N-terminal to the HECT domain. The human HECT E3 family consists of 28 members of which 15 members can be categorized into two subfamilies based on commonalities in the N-terminal domains (Fig. 4). The human NEDD4 subfamily, characterized by the presence of WW and C2 domains, has nine members and is the most prominent and well studied of the two families. The other family is the HERC E3 ligases that consist of six members and have in common that they contain one or more regulator of chromatin condensation 1(RCC)-like domains (RLD). Yeast has five HECT E3 ligases: Rsp5, Ufd4, Hul4, Hul5, and Tom1. Rsp5 is a member of the NEDD4 family, whereas the other four yeast HECT E3s do not belong to any family. Rsp5 is also the only HECT E3 that is essential for the viability of the yeast. Each of the members of the “other” human HECT E3s lack WW or RLD domains and have a distinct variety of N-terminal domains (Fig. 4).

### NEDD4 subfamily

The NEDD4 subfamily is the largest and best characterized family of the HECT E3s. The N-terminal C2 domain is defined as a Ca^2+^ and phospholipid binder [35]. Consistent with observed NEDD4 ligase functions, C2 domains are known for targeting their proteins to phospholipid membranes [36]. The C2 domain can also bind to substrate proteins to target them for ubiquitination [37, 38]. In some NEDD4 E3s, the C2 domain is involved in regulating the activity of the HECT domain, as it is capable of binding to the HECT domain, thereby folding the E3 into an auto-inhibitory conformation [39]. The NEDD4 subfamily can contain between two and four WW domains. The WW domains are responsible for the recognition of substrates [40] and have also been found to form intramolecular interactions with the HECT domain of the E3s [41].

### HERC subfamily

The HERC subfamily is characterized by having one or more RCC-like domains (RLDs), an effector protein domain that was first identified in RCC1 [42]. In humans, the HERC subfamily comprises six members, which can further be organized into two large and four small HERCs (Fig. 4). HERC1 and HERC2 are large HERCs that contain two and three RLDs, respectively. The small HERCs contain only one RLD. RLDs have dual functions as one side of the domain acts as a guanine nucleotide exchange factor (GEF) for the small GTPase Ran and the opposite side interacts with chromatin through histones H2A and H2B [43, 44]. Through their interactions with histones, HERC E3 ligases participate in various processes at the chromatin and in the nucleus (reviewed in [45]).

### “Other” HECT E3s

E6AP, the founding member of the family of HECT E3 ligases, is profoundly impactful on the regulation of the cell and has been extensively studied. Located at the N terminus (residues 24–87), E6AP harbors a zinc-binding fold called the AZUL (amino-terminal Zn-finger of Ube3a ligase) domain of which mutations have been associated with Angelman syndrome [46]. Another notable feature of the E6AP protein is an LxxLL motif (where x denotes any residue) located in the center of the protein (residues 379–395), which is the binding site of E6 [47] (for details, see “adaptor proteins and co-activators”). Although not studied as thoroughly as E6AP, the members HUWE1, UBR5, TRIP12, and HACE1 have also been subject to a significant amount of research (reviewed in [17]). HUWE1 is a giant 4374 amino-acid residue protein that contains a WWE domain, a BH3 domain, an UBA domain, and Armadillo repeats (ARM). HUWE1 naturally targets the p53 tumor suppressor for degradation, in contrast to E6AP which only targets p53 once it is hijacked by the viral E6 protein [48]. TRIP12 has also been implicated in the regulation of p53; however, this regulation is indirect through the targeting of p14ARF, a key regulator of p53 [49]. Another commonality between TRIP12 and HUWE1 is the presence of a WWE domain and Armadillo repeats. Other domains shared among this family of HECT E3s are the UBA domain (UBR5 and HUWE1), Ankyrin repeats (HACE1 and HECTD1), and IQ motifs (UBE3B and UBE3C).

## Substrate recruitment and catalytic activity regulation

### Adaptor proteins and co-activators

A prominent mechanism that regulates the substrate-specific conjugation of ubiquitin is the recruitment of HECT E3s to their substrates by adaptor proteins. Assuming that the HECT E3 is in an active state, adaptors proteins can recruit the E3 to its ubiquitination substrate, thereby contributing to the ubiquitination activity and substrate specificity. HECT E3 adaptors associate with the E3 ligases by binding to domains in the N-terminal regions or to regions within the HECT domain.

**Fig. 5:**
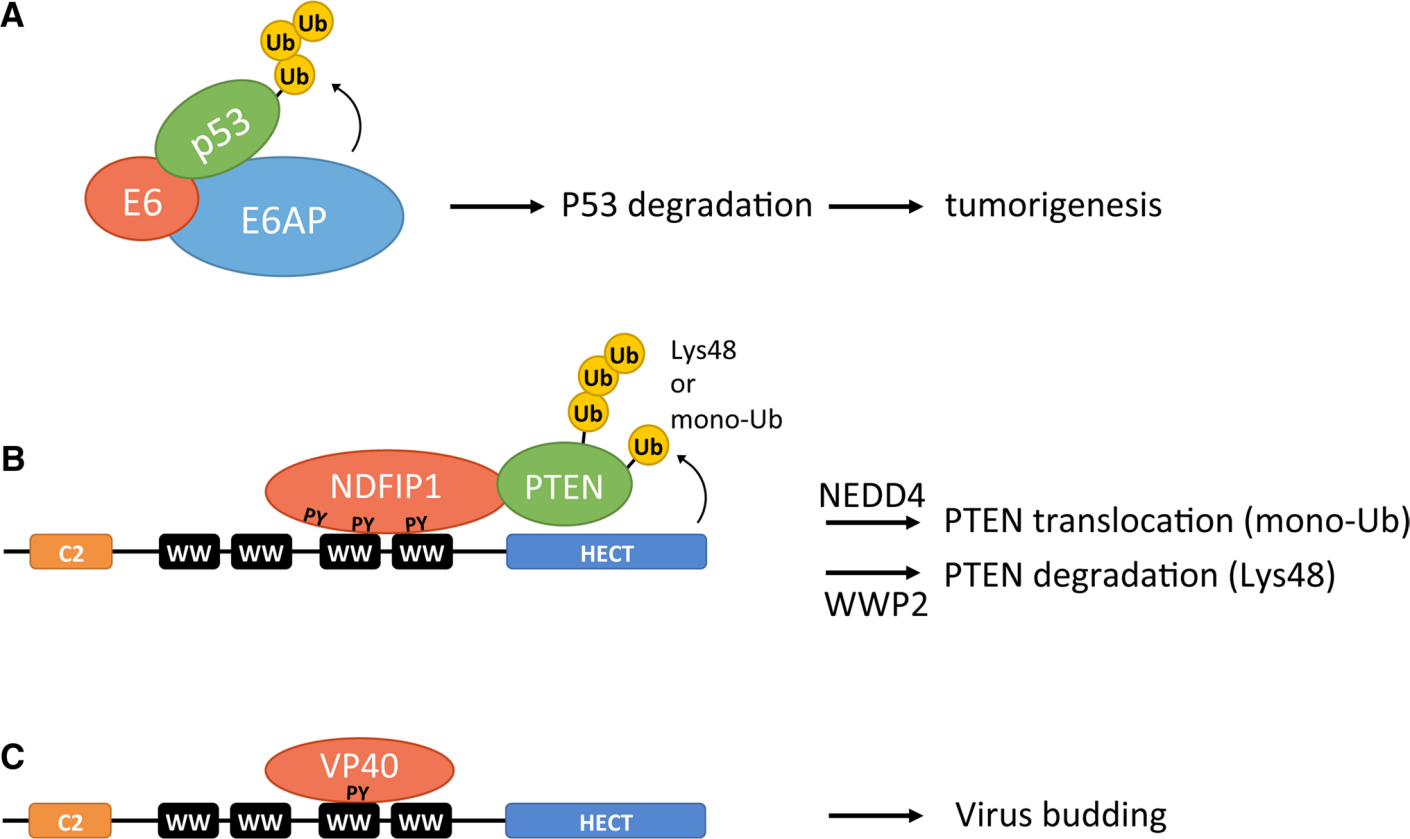
Modulation of HECT E3 ligase function by adaptor proteins and co-activators. **a** Binding of the HPV E6 protein to a conserved sequence (LxxLL motif, not shown) in the N-terminal domain of E6AP allows the E6/E6AP complex to recruit the tumor suppressor protein p53. E6AP-dependent ubiquitination of p53 targets the protein for proteasomal degradation, thereby promoting HPV-induced cervical carcinogenesis. Not shown are the other targets inactivated by the E6/E6AP complex whose degradation may contribute to carcinogenesis. **b** NDFIP1, a PY motif-containing adaptor, recruits HECT E3 ligases of the NEDD4 subfamily to their substrate. In response to ischemia, NDFIP1 recruits NEDD4 to mono-ubiquitinate PTEN, resulting in translocation of PTEN into the nucleus. Recruitment of WWP2 HECT E3 ligase by the NDFIP1 adaptor results in poly-ubiquitination of PTEN, thereby targeting PTEN for proteasomal degradation. **c** The viral VP40 protein hijacks NEDD4.1 thereby promoting ubiquitination of viral proteins and stimulating the viral budding process

A classic example of a HECT adaptor is the viral protein E6, which was the protein that led to the discovery of the HECT E3 ligases [6–8]. E6 is one of the two viral proteins expressed in HPV (human papilloma virus)-positive cervical carcinomas, the other being E7, which also utilizes the cell proteasomal system to inactivate its targets [50]. The two viral proteins each have an enormous impact on host gene expression patterns, and their oncogenic role is mainly attributed to their inactivation of tumor suppressors p53 and retinoblastoma (pRb) [7, 11, 51, 52]. The E7 oncoprotein is thought to target the pRb tumor suppressor using the cullin 2 ubiquitin ligase complex; however, this mechanism remains controversial [53, 54]. With regards to the E6 oncoprotein, it is clear that it hijacks E6AP by binding to the LxxLL motif located in the N-terminal domain of E6AP and utilizes the activity of its HECT domain to ubiquitinate p53, thereby marking it for degradation by the 26S proteasome [47] (Fig. 5a). Recent structural analysis has revealed that the binding pocket for the LxxLL motif on E6 is formed by two zinc domains and a linker helix [55]. Other than redirecting E6AP substrate specificity to ubiquitinate p53, the E6/E6AP complex also ubiquitinates other cellular proteins, the degradation of which contributes to E6-induced cellular immortalization or transformation [56]. These include the TERT (telomerase reverse transcriptase) gene repressor NFX1-91, resulting in increased telomerase activity [57, 58], E6TP1 (E6 targeted protein 1) [59, 60], MCM7(mini chromosome maintenance protein 7) [61], and BAK (Bcl-2 homologous antagonist killer) [62]. Consistent with this, knockout studies of E6 and E6AP have shown that E6 relies virtually exclusively on E6AP to alter the cellular gene expression [63].

The alterations that E6 binding creates in the substrate specificity of E6AP cannot always simply be explained by the binding of E6 to substrates. Studies have shown that p53 does not bind E6AP or the E6 adaptor in isolation from each other [64], whereas other studies showed only weak and insignificant E6–p53 interactions [65, 66]. Consistent with this, a recent analysis of the crystal structure of the E6/E6AP/p53 ternary complex showed that the p53 binding cleft on E6 is formed upon binding of the LxxLL peptide [67]. Other substrates recruited to the E6/E6AP complex by E6 show that the viral protein does function in a manner expected from an adaptor protein. For example, the E6 proteins of ‘high-risk’ HPV have a C-terminal PDZ (named for the proteins PSD95, DLG, and ZO1) domain-binding motif (PBM) through which they bind PDZ-domain-containing substrates irrespective of whether the E6 is in complex with E6AP [68]. Nineteen PDZ-domain-containing proteins have been confirmed as binding partners of E6, of which several are known as tumor suppressors [69, 70]. Experiments with cell and mouse models show that the PBM is important for carcinogenesis [71–74] and that this is reliant upon proteolytic targeting of PDZ-domain-containing substrates by the E6/E6AP complex [75–77]. This is consistent with the observation that all cancer-causing HPV types contain a PBM, whereas low-risk HPV types often do not contain a PBM (reviewed in [70]).

The binding of E6 to E6AP does not only affect its substrate recognition, but also the catalytic activity of its HECT domain is increased. Consequently, its regular substrate proteins are also increasingly ubiquitinated by the E6/E6AP complex [78]. Notably, a similar allosteric interaction, which enhances E6AP activity, has been seen with HERC2. HERC2, another HECT E3, binds to E6AP in a region on the N terminus (residues 150–200) and thereby increases the catalytic activity of E6AP [79]. Together these studies show that E6 is a versatile adaptor that recruits substrates to the E6AP complex and additionally increases the catalytic activity of the HECT domain through allosteric interactions.

It should not come as a surprise that viruses utilize adaptors to control HECT E3 ligases, as the cell also commonly uses them to regulate HECT E3s. Some of the most extensively studied cellular adaptors are those that interact with the WW domains of the NEDD4 subfamily. Adaptors of NEDD4 typically contain PY motifs (PPxY or LPxY, x is any residue) that interact specifically with WW domains [80, 81]. One informative example of a target that is recruited to NEDD4 E3 ligases through interaction with PY motif-containing adaptors is PTEN (phosphatase and tensin homolog). PTEN is a plasma membrane lipid phosphatase that acts as an antagonist of the phosphatidylinositol-3-kinase (PI3K) signaling pathway by dephosphorylating the second messenger phosphatidylinositol 3,4,5-trisphosphate (PIP_3_) to phosphatidylinositol 4,5-bisphosphate (PIP_2_) [82]. Through its role in phosphatidylinositol homeostasis, PTEN is recognized as a pivotal tumor suppressor and regulator of cellular processes including proliferation, survival, and migration [83]. Several PY motif adaptors have been found to mediate PTEN ubiquitination including NDFIP1 (Nedd4 family-interacting protein 1) and NDFIP2 and more recently NUMB. The NDFIP adaptors can recruit various NEDD4 E3s to target PTEN either for degradation or nuclear translocation [84–86] (Fig. 5b). In response to ischemia, NDFIP1 recruits NEDD4.1 or NEDD4.2 to mono-ubiquitinate PTEN, which leads to translocation of PTEN to the nucleus [84], where it controls processes not related to PIP_3_ hydrolysis such as chromosome integrity and cell cycle progression [83]. By contrast, NDFIP-mediated recruitment of PTEN to the HECT E3 WWP2 results in PTEN poly-ubiquitination and its subsequent degradation [85]. These observations suggest that the HECT E3 that is recruited by the adaptor determines the outcome for the substrate. WWP2 likely ubiquitinates PTEN with Lys48 and/or Lys11-linked chains that are commonly associated with proteasomal degradation, whereas NEDD4.1 has been shown to mono-ubiquitinate PTEN [86], a modification often associated with non-proteolytic functions. Intriguingly, recruitment of NEDD4.1 by a different adaptor, NUMB, results in PTEN poly-ubiquitination and degradation [87], suggesting that the adaptor may also influence the type of ubiquitin chain catalyzed by the HECT E3. Another NEDD4 member regulated by NDFIP1 is ITCH, an E3 ligase that targets the transcription factor JunB for degradation in a way reminiscent to that of the recruitment of WWP2 to the substrate PTEN. Indeed in the absence of NDFIP1, ITCH cannot bind JunB to ubiquitinate it, resulting in the accumulation of JunB. Consistent with this, the absence of NDFIP1 has been shown to cause inflammation in mice as a result of JunB accumulation [88].

Interactions of PY-containing adaptors with the WW domains of HECT E3s are also seen for viral proteins (Fig. 5c). The viral protein VP40, which is found in the filoviruses Ebola (eVP40) and Marburg (mVP40), contains a proline-rich PPxY motif that it utilizes to hijack NEDD4.1 through binding of its third WW domain [89, 90]. The release of viral particles is dependent on the binding of VP40 to NEDD4.1, thereby hijacking its ubiquitination activity to mediate virus budding [91]. A recent study also showed that the VP40 proteins hijack ITCH to regulate the budding process [92]. Given the HPV viral protein E6 hijacking E6AP to suppress p53 and VP40 hijacking NEDD4 family E3s to mediate virus budding, it is ostensible that HECT E3s are desirable targets which viruses can use to control their host cells. Hence, inhibiting these HECT E3–viral protein interactions, for example, with small molecules can be of therapeutic relevance. Indeed various compounds have been found that inhibit the interaction between the Ebola and Marburg virus VP40 proteins and NEDD4 HECT E3s [93]. Although these compounds may inhibit interactions of NEDD4 with any PPxY motif-containing protein, they are still attractive potential antiviral agents. Screens for small molecule inhibitors have also been successfully done to find compounds that inhibit the interaction between E6 and E6AP [94]. Several of these inhibitory compounds were shown to block p53 degradation in HPV-infected cells. Further study of these compounds may lead to the development of beneficial therapeutics.

### Regulation by recruitment of E2-conjugating enzymes

**Fig. 6:**
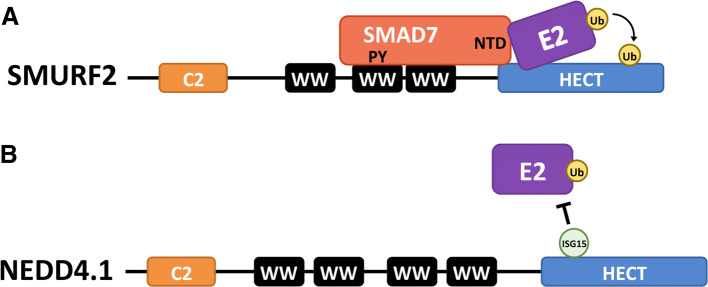
Regulation of E3-E2 interactions. **a** Binding of SMAD7 to SMURF2, through interaction of its PY motifs with one of the WW domains in SMURF2, not only relieves the auto-inhibitory conformation of the HECT E3 (see Fig. 7b) but also stimulates E2 binding: the amino-terminal domain (NTD) of SMAD7 contacts both the SMURF HECT and the E2, thereby enhancing E2 binding and ubiquitin ligase activity. **b** Non-covalent binding of interferon-stimulated gene 15 (ISG15) to NEDD4.1 blocks interaction of the HECT E3 with its E2-conjugating enzyme, resulting in reduced E3 ligase activity

HECT ubiquitination activity is also regulated at the level of recruiting E2-conjugating proteins. SMAD7 functions as an adaptor for SMURF1 and SMURF2 by recruiting these HECT E3s to their substrate the TGF-β receptor [95]. Similar to the mechanism of NDFIP1- and NDFIP2-mediated recruitment described previously, SMAD7 has PY motifs that interact with the WW domains of the SMURF E3s. In addition to its function as an adaptor, SMAD7 also has a function to activate the HECT domain. SMAD7 recruits UbcH7, the E2-conjugating enzyme of SMURF2, thereby enhancing the ubiquitin ligase activity of the E3 [96]. To facilitate E2–E3 interaction, SMAD7 binds the HECT domain of SMURF2 and binds UbcH7 with its N-terminal domain (Fig. 6a). Analysis of the E2-binding domain of SMURF2 suggested that SMURF2 has an inherent low affinity for its E2-conjugating enzyme. This low E2–E3 binding affinity suggests that the E3 enzyme is dependent on other proteins for optimal interaction with its E2-conjugating enzyme and thus its ubiquitination activity.

In addition to the recruitment of E2s by adaptors, inhibitors that prevent the HECT E3s from interacting with E2-conjugating enzymes can also regulate HECT activity. The interaction of NEDD4.1 with its E2-conjugating enzyme is negatively regulated by interactions with ISG15, an ubiquitin-like protein. The resulting decrease in NEDD4 E3 ligase activity causes a reduced ubiquitination of the Ebola virus VP40 (discussed in the previous section), thereby blocking viral budding [97, 98] (Fig. 6b). ISG15 is an interferon-stimulated gene that can be present in the cell in free form or covalently bound to a substrate as a result of an enzymatic process called ISGylation [99]. ISGylation of NEDD4.1 is not necessary to obtain the inhibitory effect on NEDD4.1; non-covalent interactions of ISG15 with NEDD4.1 are sufficient to prevent the E2–E3 interaction.

### Inter- and intramolecular interactions of HECT E3 ligases

HECT E3 ligases are often regulated by intramolecular (i.e., within the same molecule) or intermolecular (i.e., with other identical proteins) interactions. Through these intra- and intermolecular mechanisms, the HECT E3s can control the activity of their own catalytic domain. Often these mechanisms determine the default state of the HECT protein and, thus, regulatory proteins that change these inter- and intramolecular interactions can control the activity of the HECT E3.

#### Interactions of N-terminal domains with the HECT domain

**Fig. 7:**
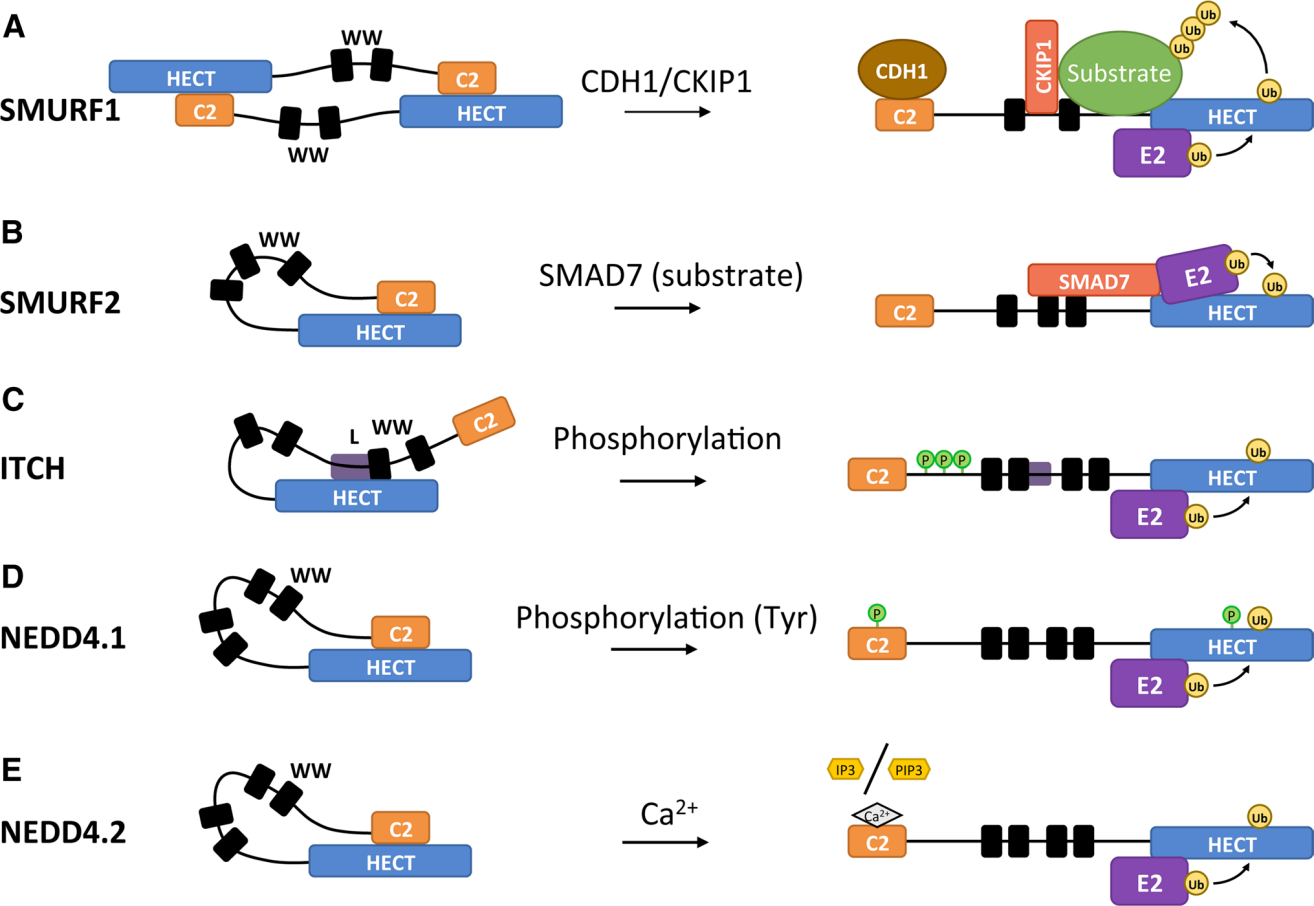
Modes of relief of auto-inhibitory interactions of the NEDD4 subfamily. Various mechanisms are known to relieve auto-inhibitory interactions between HECT and C2/WW domains. SMURF1 forms an auto-inhibited homodimer through intermolecular interaction of the C2 domain of one monomer with the HECT domain of the other (**a**), while in all other known NEDD4 subfamily members the auto-inhibitory conformation is mediated by intramolecular interactions involving C2 or WW domains and the HECT domain (**b**–**e**). **a** Allosteric interactions of various adaptor proteins with SMURF1 disrupt the SMURF1 auto-inhibited homodimer and promote substrate binding. CDH1 (Cadherin 1) and CKIP1 (Casein kinase-2 interacting protein 1) may work sequentially: interaction of CDH1 with the C2 domain disrupts the SMURF1 homodimer, subsequent binding of CKIP1 to the linker region between the WW domains promotes binding and ubiquitination of SMURF1 substrates. **b** Binding of SMAD7 to WW domains of SMURF2 relieves auto-inhibition and enhances E2 binding, thus stimulating E3 ligase activity (see also Fig. 6a). Serine/threonine phosphorylation of ITCH in a proline-rich region between the C2 and WW domains (**c**) or tyrosine phosphorylation of the NEDD4.1 C2 domain (**d**) relieves auto-inhibition. **e** The increase in Ca^2+^ concentration in the cell disfavors the NEDD4.2 C2-HECT domain contacts, thereby relieving the auto-inhibitory conformation. In the presence of Ca^2+^, the C2 domain can bind PIP_2_ (phosphatidylinositol 4,5-bisphosphate) head groups in the membrane or IP_3_ (inositol 1,4,4-triphosphate) molecules in the cytoplasm, which may in turn determine the intracellular localization of the HECT E3 ligase. *L* linker region connecting the WW2 and WW3 domains. For details, see text

Some HECT E3s can arrange themselves into inactive conformations by interactions of the N-terminal region with the HECT domain. Several of the WW and C2 domains of the NEDD4 E3s can interact with their corresponding HECT domains, thereby blocking access to the catalytic site. Due to these conformations, it is common for NEDD4 E3 ligases to have a default state of auto-inhibition. Although the domain architecture of the NEDD4 family is similar, the mechanisms of auto-inhibition vary [39, 100, 101]. Two exemplary mechanisms of auto-inhibition can be found in ITCH and SMURF2 (Fig. 7). The auto-inhibited state of ITCH is acquired through the binding of its WW domain region to its HECT domain (Fig. 7c) [41]. Recent structural analysis has revealed further details of the auto-inhibitory conformation of ITCH: the interface of the auto-inhibited ITCH involves the second WW domain (WW2) and a linker region connecting WW2 and WW3 [102]. The WW3 domain may still be relevant for auto-inhibition as previous research established that mutations in either WW2 or WW3 resulted in activation of ITCH [101]. The importance of the WW2–WW3 linker region in regulating E3 ligase activity was recently confirmed for WWP2, a HECT closely related to ITCH [103]. Indeed, Chen et al. establish that a linker region connecting the WW2 and WW3 domains is necessary to lock the E3 in its auto-inhibitory conformation. The SMURF2 inhibitory state is acquired similarly, but instead of the WW domain region it is its C2 domain that binds to the HECT domain (Fig. 7b) [39]. Similar to SMURF2, interactions between the C2 and HECT domains also inhibit SMURF1 (Fig. 7a). In contrast, SMURF1 inhibitory interactions are not intramolecular. SMURF1 is not able to fold into an intramolecular auto-inhibitory state likely due to the shorter nature of the protein. Instead, the C2-HECT domain auto-inhibitory interactions of SMURF1 proteins are facilitated by the formation of homodimers [104].

The regulatory mechanisms that the cell uses to control HECT E3s often revolve around relieving previously discussed auto-inhibitory interactions. Intuitively, a wide variety of mechanisms can be envisioned that relieve these auto-inhibitory and homo-oligomeric interactions. The previously mentioned examples of ITCH, SMURF1, and SMURF2 inhibitions also provide good examples of the various mechanisms that relieve inhibition (Fig. 7). SMURF2 auto-inhibition is relieved by the binding of its substrate SMAD7 to the WW domain adjacent to the HECT domain (Fig. 7b) [105]. Disruption of the SMURF1 homodimer is achieved by allosteric interactions of several adaptor proteins including CKIP (casein kinase-interacting protein), which binds to the WW domain [106]; CDH1 (Cadherin 1), which binds to the C2 domain [104]; or CCM2 (cerebral cavernous malformations 2), which binds to the HECT domain (Fig. 7a) [107]. Recent evidence suggests that CKIP and CDH1 have different roles in SMURF1 activation [108]. While CDH1 is required for disruption of the auto-inhibited SMURF1 homodimer, CKIP1 promotes binding and ubiquitination of SMURF1 substrates. The auto-inhibitory conformation of ITCH is relieved by phosphorylation (Fig. 7c). In this instance, ITCH is phosphorylated at residues S199, S232, and T222, all of which are located in a proline-rich region (PRR) between the C2 and WW domains [101]. PY motif-containing interactors such as NDFIP1 and NDFIP2 can also release auto-inhibition through their interactions with WW domains [102]. To release auto-inhibitory conformations involving the WW-linker-binding interface such as in ITCH, the PY motif-containing co-activators are likely to rely on multiple PY–WW interactions able to disrupt the auto-inhibitory WW-linker to HECT binding. This linker region may also be directly targeted to relieve auto-inhibition. The WW2–WW3 linker contains tyrosine phosphorylation sites, and experiments mimicking phosphorylation of these sites in WWP2 showed increased activity of the HECT [102]. The linker regions connecting the various N-terminal domains of HECT E3 ligases may have a more impactful role on HECT regulation than previously anticipated. Chen et al. [103] discuss a linker region C-terminal of the WW1 domain in NEDD4.1 that has an α-helical structure similar to the WW2–WW3 linker in WWP2. Indeed deletion of this linker region in NEDD4.1 resulted in increased auto-ubiquitination activity. In addition, tyrosine phosphorylation has been shown to relieve the auto-inhibitory interactions of NEDD4.1 (Fig. 7d). NEDD4.1 is phosphorylated at a tyrosine residue in the C2 and in the HECT domain by c-Src kinase [13].

NEDD4.2 is regulated by its C2 domain through another distinct mechanism (Fig. 7e). The auto-inhibited form of NEDD4.2 is stabilized by the interaction of its C2 domain with its HECT domain. Increased Ca^2+^ concentration in the cell leads to the activation of NEDD4.2 by disrupting this auto-inhibitory conformation [109]. The HECT-binding site on the C2 domain is shared with the Ca^2+^ binding site, suggesting that the Ca^2+^ interactions cause repulsion of the HECT domain. In addition to the activation of NEDD4.2, these interactions may also be involved in the localization of the HECT E3 ligase. NEDD4.2 can be relocated through interactions of its C2 domain with the head group of phosphatidylinositol 4,5-bisphosphate (PIP_2_) and its second messenger inositol 1,4,5-trisphosphate (IP_3_), which is the soluble version of the PIP_2_ head group. The interactions of PIP_2_ and IP_3_ are facilitated by Ca^2+^ ions that form a bridge to the C2 domain. PIP_2_ prefers localization at the cell membrane, whereas IP_3_ prefers to diffuse throughout the cytoplasm. The PIP_2_/IP_3_ ratio can determine the distribution of NEDD4.2 as localization of the E3 depends on which of the two molecules it associates with [109]. These results may suggest phospholipase C (PLC) as an important upstream regulator of NEDD4.2 as PLC triggers the increase of Ca^2+^ in the cytoplasm and controls the ratio of PIP_2_/IP_3_ through the hydrolysis of PIP_2_. Since all NEDD4 ligases contain a C2 domain, the discussed regulation through Ca^2+^ may be more common among this HECT E3 family.

#### Ubiquitin-binding domain controlled oligomerization

Formation of intermolecular interactions between identical HECT E3 ligases has also been studied in the NEDD4 yeast ortholog Rsp5. Similar to SMURF1, Rsp5 does not appear to be inhibited by intramolecular interactions [39], but rather through the formation of Rsp5 oligomers [110, 111]. However, the inhibitory interactions of Rsp5 are substantially different from the previously discussed inhibitory interactions of NEDD4 ligases between the C2 and HECT domains. Rsp5 is proposed to utilize an oligomerization interface in its HECT domain to form inhibitory trimers. Interestingly, the experiments of Attali et al. suggest that the accessibility of this trimerization interface is controlled by the ubiquitination of specific lysine residues in an α-helical segment N-terminal to the HECT domain, called the α1-helix [33]. The HECT ubiquitin-binding domain (HECT-UBD), also known as the ‘exosite’ [112, 113], has an important role in this regulatory mechanism as its pull on the ubiquitin conjugated α1-helix is suggested to lead to a conformational change that exposes the trimerization interface [110]. The resulting trimerization shuts down the catalytic activity of the HECT domain. Conservation analysis of the α1-helix domain suggested that this oligomerization mechanism is also prevalent among human NEDD4 ligases. Experiments using NEDD4.1 with a ubiquitin-fused α1-helix and a NEDD4.1^K523,525R^ mutant that cannot be ubiquitinated at its α1-helix are consistent with this [110]. Ubiquitin-fused NEDD4.1 results in its oligomerization and diminished ubiquitination activity, whereas NEDD4.1^K523,525R^ did not form oligomers and exhibited increased activity. Thus, ubiquitination of the α1-helix may be an important regulatory mechanism to control the activity of various NEDD4 ligases. As NEDD4.1 auto-ubiquitinates the α1-helix, a deubiquitinating enzyme may control the activity of the E3. It may be that the α1-helix ubiquitin-mediated oligomerization is as common as the C2-HECT domain oligomerization, where the former is controlled by the ubiquitin status of the α1-helix and the latter by phosphorylation or Ca^2^ binding.

#### Activating E6AP–E6AP interactions

Oligomerization may also play a role in the activation of HECT E3s rather than their inhibition. E6AP trimerization has been reported to promote its ubiquitin ligase activity [114]. Early evidence for cooperative E6AP–E6AP interactions were already suggested, because E6AP was only found to efficiently auto-ubiquitinate in the presence of other E6AP enzymes, suggesting intermolecular transfer of ubiquitin [115]. In support of an oligomeric structure of E6AP, crystallographic analysis of its HECT domain revealed a trimeric arrangement that is stabilized through N-lobe/N-lobe interactions [31]. However, the trimeric form of E6AP was initially dismissed as the HECT domain construct is monomeric in solution and a mutation in the trimerization interface (F727A) did not affect the ability of the enzyme to transfer ubiquitin from E2 to its active site [31]. Rather than forming oligomeric complexes, the interactions between E6AP were proposed to be transient and the trimeric form of the E6AP HECT domain in the crystal was attributed to a crystallization artifact. However, recent biochemical experiments showed that GST (glutathione *S*-transferase)-fused E6AP has an increased poly-ubiquitin chain formation in vitro compared to the un-fused E6AP. These observations suggested that GST–GST interactions [116] promote E6AP oligomerization resulting in increased activity, which prompted further research into the role of E6AP oligomerization [114]. Kinetic and biophysical experiments with the un-fused, full-length, E6AP provided stronger evidence for an activating role of E6AP oligomerization, as E6AP trimers were found to be more active than their monomeric counterparts. In support of this, mutation of a key residue in the trimerization interface of E6AP, F727, which binds to a hydrophobic pocket of the adjacent subunit, substantially decreased the catalytic activity of E6AP using polyubiquitin chain formation as a functional readout. The E6AP trimer harbors a second subunit interface important for the stabilization of the trimer [31, 114, 117]. The second interface is located further from the catalytic site. Interactions at this second interface were shown to be regulated by tyrosine phosphorylation [117], which is discussed more elaborately in the chapter of post-translational modifications. Mutation of key residues at this second subunit interface also reduce the catalytic activity of E6AP [114], further supporting the notion that the fully active form of E6AP is a trimer.

Remarkably, none of the other crystallized HECT domains forms a crystallographic trimer (reviewed in [16, 118]), suggesting that trimerization is a unique feature of the E6AP HECT domain. However, the crystallized E6AP HECT construct lacks an α-helix segment N-terminal of the HECT domain (α-1 helix; previously discussed to regulate Rsp5 oligomerization) that is present in all other crystallized HECT domain constructs. In these structures the α-1 helix shields the hydrophobic pocket otherwise occupied by F727, suggesting that the presence of the α-1 helix in these HECT domain constructs may prevent trimerization. In support of a role of the α-1 helix in regulating E6AP oligomerization, Ronchi et al. [114] showed that the addition of a peptide corresponding to the α-1 helix promoted the dissociation of the E6AP trimer and strongly reduced its E3 ligase activity in vitro. It is remarkable that trimerization of E6AP and Rsp5 seems to have opposing outcomes, namely activation of the former and inactivation of the latter. Although modeling has suggested that Rsp5 (and possibly other NEDD4 family members) uses similar interfaces as E6AP to form trimers [110], further structural analysis is required to confirm this hypothesis. In addition, there is currently no structural information available on the full-length proteins. Therefore, it remains unclear what the contribution is of the regions N-terminal to the HECT domain as well as if/how intra- and intermolecular interactions impact E3 ligase function in the proposed trimers.

#### HUWE1 oligomerization and auto-inhibition

HUWE1 is a HECT E3 that can be down-regulated through a combination of oligomerization and auto-inhibitory interactions [119]. HUWE1 forms an auto-inhibitory homodimer where both inter- and intramolecular interactions are involved in the inhibition. In this case, a crystal structure of the C-terminal domain of HUWE1 revealed an asymmetric dimer that is stabilized through hydrophobic interactions mediated by a region adjacent to the HECT domain. HUWE1 counteracts its own inhibition through an intramolecular interaction with a segment located 50 residues upstream of the dimer-binding region. The HUWE1 inhibitor and tumor suppressor p14ARF have been shown to bind and disable this ‘activation segment’, thereby promoting inhibitory dimerization. The involvement of the p14ARF inhibitor suggests that, in contrast to the previously discussed oligomerization mechanisms, the inhibitory conformation of HUWE1 is not its default state. Structural analysis of the asymmetry of the HUWE1 dimer is intriguing, as it appears to affect the activity status of the two HECT domains. The HECT activity of one subunit in the dimer is impaired by a conformational lock of the C-lobe and blocked access to the catalytic site through interactions with the other subunit. This latter subunit may be inhibited through allosteric interactions in the dimer; however, there is no indication that the flexibility of the C-lobe of this subunit is restricted, so its HECT domain may still be catalytically relevant (for details see [16, 119]). It appears that HUWE1 has a unique oligomerization mechanism in contrast to the previously discussed more common oligomerization mechanisms.

### Post-translational modifications of HECT E3 ligases

Various post-translational modifications of HECT E3 ligases are known to influence catalytic activity. Phosphorylation and ubiquitin-like modifications are types of post-translational modifications that commonly regulate various HECT E3s. Post-translational modifications can influence HECT activity by causing conformational changes in the E3 ligases or by influencing interactions of HECT E3s with adaptors or other regulatory proteins.

#### Phosphorylation

As previously discussed, phosphorylation of a PRR of ITCH can activate the HECT domain by relieving ITCH from its auto-inhibitory fold (see Fig. 7c). More specifically, ITCH is phosphorylated on three residues (S199, T222, S232) of a PPR residing in between the C2 and the first WW domain by the JNK1 serine/threonine kinase [101, 120]. Recruitment of JNK1 to ITCH is facilitated by an interaction of a D domain-like sequence within the N-lobe of the HECT domain. D domains consist of a group of basic residues followed by a cluster of hydrophobic residues and have been shown to recruit MAP kinases [121]. Phosphorylation of the PRR region weakens the interaction between the WW domain region and the HECT domain, thereby disrupting the auto-inhibitory conformation of ITCH [101]. The PRR of ITCH is the only known phosphorylation site of JNK1 in HECT E3s so this mechanism may be unique to ITCH.

NEDD4.1 is another example where the auto-inhibition of a HECT E3 is relieved by phosphorylation (Fig. 7d). The mechanism varies from the previously discussed type of NEDD4 family phosphorylation in that NEDD4.1 is phosphorylated at tyrosine residues in the C2 (Y43) and HECT domain (Y585), and this phosphorylation is catalyzed by the c-Src kinase [13]. The phosphorylated C2 and HECT domains of NEDD4.1 do not form the auto-inhibitory conformation most probably due to electrostatic repulsions caused by the phosphate groups. SYK-mediated tyrosine phosphorylation of HUWE1 is another example of how phosphorylation can disrupt auto-inhibition [122]. Unlike the previously discussed mechanism where addition of a phosphate moiety disrupts auto-inhibition, phosphorylation of HUWE1 causes the disassociation of HUWE1 from its inhibitor p14ARF. As discussed previously, the release of p14ARF enables HUWE1 to form an intramolecular conformation that counteracts the inhibitory dimer.

One of the earliest cases reported of a HECT E3 regulated by phosphorylation is that of NEDD4.2 [123]. NEDD4.2 is phosphorylated by the kinase SGK1 (serum/glucocorticoid-regulated kinase 1) which utilizes a PY motif that binds to the E3s WW domains [124]. Serine phosphorylation (S468) of NEDD4.2 promotes recruitment of the adaptor protein 14-3-3 to the phosphorylated NEDD4.2, thereby reducing the interaction of NEDD4.2 with its natural substrate ENaC (Epithelial Na^+^ Channel), leading to increased ENaC surface expression.

Phosphorylation has also been shown to negatively regulate the E3 ligase activity of E6AP [117]. E6AP is tyrosine phosphorylated by c-Abl at residue 636 within its HECT domain. Mutation analysis of Y636 suggests that this residue controls the E3 ligase activity in a substrate-specific manner. This substrate specificity was shown for the E6AP phosphorylation resistant mutant Y636F, which has impaired E3 ligase activity towards the substrate hHR23A (human homolog of Rad23), although the ubiquitination of p53 in the presence of HPV E6 protein remained similar to that of wild-type E6AP. Analysis of the E6AP crystallographic trimer suggested that the Y636 is present in the second subunit interface important for stabilization of E6AP oligomers. Since the oligomerization of E6AP has been shown to promote its E3 ligase activity, it is conceivable that Y636 phosphorylation regulates E6AP by deterring its ability to oligomerize. However, direct evidence for this is lacking.

Mutants of E6AP that influence its phosphorylation are not exclusive to those introduced with gene editing. A mutation of threonine 485 to alanine in E6AP was identified in a whole-exome sequencing study aimed at identifying de novo mutations linked to autism [125]. Detailed analysis of the T485A mutation led to the discovery that T485 is phosphorylated by PKA (protein kinase A) as a mechanism to regulate E6AP E3 ligase activity [126]. The substitution of threonine for an alanine makes it impossible to phosphorylate the residue and thereby disables phosphorylation control of E6AP. In HEK293T cells, it was shown that the E6AP^T485A^ mutant actively auto-ubiquitinates, whereas a T485E mutant that mimics phosphorylated E6AP was inhibited for auto-ubiquitination. In addition to reducing auto-ubiquitination, phosphorylated E6AP was also shown to have repressed E3 ligase activity towards substrates such as hHR23A using in vitro analysis with the same mutants. Interestingly, the phosphorylation at T485 not only affects E3 ligase activity but also the ability of E6AP to self-associate. While wild-type E6AP and the phospho-mimetic T485E mutant showed no self-association, the T485A mutant was able to interact with itself [126]. Whether the observed self-association of the T485A mutant is related to the crystallographic E6AP trimer remains to be elucidated. A recent follow-up study on the E6AP^T485A^ mutant revealed that its expression results in increased Wnt (wingless-type MMTV integration site family member) signaling through the ubiquitination of several proteasome subunits that are part of a distinct proteasome subdomain [127]. The ubiquitination of proteasome subunits by E6AP reduces proteasome subunit abundance and activity [128, 129], resulting in the accumulation of stable β-catenin in the nucleus and subsequent activation of the Wnt signaling pathway [127]. Together, the studies on the phospho-resistant E6AP^T485A^ show how the phosphorylation of one residue in a HECT E3 can control crucial cellular processes.

#### Ubiquitination/deubiquitination

Ubiquitination is another PTM that can regulate HECT E3 ligases. HECT E3 ligases may be ubiquitinated by other E3 ligases or ubiquitinate themselves in a process called auto-ubiquitination. Often the ubiquitination of HECT E3 ligases functions as a mechanism of down-regulation by marking them for proteasomal degradation [130]. The regulatory effects of ubiquitin on HECT E3s are often controlled at the level of deubiquitination.

One HECT E3 that is negatively regulated by ubiquitination is TRIP12. TRIP12 is ubiquitinated and thereby targeted for proteasomal degradation. The control of this regulatory degradation has been attributed to the DUB (deubiquitinating enzyme) USP7 [131]. USP7 associates with TRIP12 and stabilizes it by deubiquitinating the HECT E3. This USP7–TRIP12 interaction has been shown to lead to the ubiquitination of the tumor suppressor p14^ARF^. Unsurprisingly, overexpression of USP7 and TRIP12 has been found to be associated with poor prognosis in cancers with aberrant expression of p14^ARF^, such as hepatocellular carcinoma (HCC) [131]. Interestingly, USP7 itself is also targeted by TRIP12 for proteasomal degradation, indicating that they are mutual substrates for each other [132]. Thus, USP7 increases the stability of TRIP12, whereas the TRIP12 does the opposite for USP7. Their effectiveness in targeting their cognate substrates relies on the homeostatic balance of the two proteins.

E6AP is another example of a HECT E3 ligase that is regulated by ubiquitination, serving as a substrate for UBR5, another HECT E3 ligase [133]. UBR5 negatively regulates E6AP abundance, and as a result the loss of UBR5 leads to higher levels and a longer half-life of E6AP. While this regulatory effect does occur in uninfected cells, most of the regulatory effects of UBR5 on E6AP are associated with HPV-infected cells. UBR5 interacts strongly with the HPV protein E6 and UBR5 impairs the ability of the E6/E6AP complex to target its substrates for degradation.

Ubiquitination by the yeast HECT E3 ligase, Rsp5 is negatively regulated through an interaction with the Ubp2 deubiquitinating enzyme [134]. When in complex with the HECT E3 ligase, Ubp2 antagonizes the ubiquitination by Rsp5 by catalyzing the opposing reaction. In this case, the ubiquitin-associated (UBA) domain-containing protein Rup1 mediates the coupling of Ubp2 to Rsp5. The UBA domain of Rup1 is not necessary for the interaction between Rsp5 and Ubp2; however, the UBA domain does stimulate Ubp2 deubiquitinating activity. The coupling of Rsp5 to the DUB is not necessarily a general negative regulator of Rsp5 ubiquitination as it may have specificity towards the substrates it deubiquitinates. This substrate specificity would allow Ubp2 to determine which Rsp5 substrates are ultimately ubiquitinated. In addition, Ubp2 may play an important role in determining the topology of ubiquitin chains catalyzed by Rsp5. Deubiquitination can limit the extension of ubiquitin chains, thereby favoring mono-ubiquitination over poly-ubiquitination of certain substrates.

#### SUMOylation

A recent study showed that the conjugation of the small ubiquitin-like modifier (SUMO) to SMURF2 regulates the activity of the SMURF2 HECT domain [15]. The SUMOylation of SMURF2 enhances its ubiquitination activity towards its target TβR1 (transforming growth factor B receptor 1), resulting in degradation and concomitant suppression of the TGF-β pathway. SUMOylation of SMURF2 is facilitated by the SUMO-E2-conjugating enzyme UBC9 and the SUMO E3 ligase PIAS3, which target distinct lysine residues of SMURF2. In a follow-up study, it was shown that SMURF2 SUMOylation suppresses the invasiveness of breast cancer organoids [135]. The mechanism through which SUMOylation regulates SMURF2 activity has not yet been determined. However, the location of the lysine residues SUMOylated by PIAS3, in the C2 domain (Lys26) and next to the C-terminal WW domain (Lys369), may suggest which regulatory mechanisms of the HECT E3 are altered. Proximity of the Lys369 SUMOylation site to the WW domains may affect the binding of these domains to the PY motifs of adaptor proteins, while SUMOylation of Lys26 may be involved in the formation or disruption of the auto-inhibitory conformations mediated by interactions between the C2 and HECT domain.

#### NEDD8 modification

Neddylation, the covalent binding of NEDD8 onto a target protein, is another ubiquitin-like modification that has been shown to regulate HECT E3 ligases. NEDD8 is 58% identical to ubiquitin and is covalently bound to its substrates in a process analogous to ubiquitination, using dedicated E1 (NAE) and E2s (Ube2M and Ube2F) for activation and transfer [136, 137]. Neddylation is a post-translational modification primarily known for regulating cullin-RING ligases (CRLs), a superfamily of RING E3 ligases [138]. The cullin subunit of the CRL complex needs to be neddylated on a single conserved lysine residue close to its RING-binding site to allow recruitment of the E2 critical for ubiquitin ligation activity [139]. Various non-CRL neddylation targets have been proposed and are being investigated including the HECT E3 SMURF1 [14, 140]. SMURF1 catalyzes its own covalent attachment of NEDD8 to activate its ubiquitin ligase activity [14]. In contrast to the specific neddylation of a single lysine residue seen in CRL neddylation, SMURF1 neddylates multiple lysine residues in various domains. Whether neddylation activates SMURF1 through promoting E2–E3 interactions similar to CRL activation or through other mechanisms remains to be discovered. In addition to SMURF1, SMURF2 has also been shown to be neddylated and this neddylation leads to SMURF2 ubiquitination and its sequential degradation [141]. Non-covalent binding of NEDD8 has also been shown to promote ubiquitin ligase activity of SMURF1 and SMURF2 [142]. The Smurf proteins contain a conserved domain that binds NEDD8, and disruption of this binding inhibits ubiquitin ligase activity of the Smurf HECT E3s [142]. A study of the other members of the NEDD4 subfamily found that ITCH, NEDL1, and NEDL2 can also catalyze auto-neddylation in addition to the Smurf E3s [143]. Exceptionally, ITCH is the only HECT that is neddylated on a single lysine residue, similar to the CRL E3s. ITCH also functions as a neddylation E3 for JunB, targeting it for sequential ubiquitination and degradation [143].

#### ISG15

The interferon inducible ISG15 is another ubiquitin-like protein that has been shown to regulate the activity of NEDD4.1. As discussed above, binding of free ISG15 to NEDD4.1 blocks the interaction of the E3 with its E2 thereby inhibiting the ubiquitin ligase (Fig. 6b) [97]. Notably, ISG15 expression is induced in response to viral infections and its inhibiting effect on NEDD4.1 is an effective counter mechanism to the virus. Similar to the NEDD8 interaction with SMURF1 and SMURF2, ISG15 binds NEDD4.1 through non-covalent interactions. It is possible that NEDD4.1 is covalently modified by ISG15 (ISGylation); however, the interaction with free ISG15 seems to be sufficient to inhibit the E3s ubiquitination activity [98]. Whether post-translational modifications with ISG15 also regulate HECT E3 ligases remains to be determined. ISGylation has been shown to regulate non-HECT E3 ligases such as Parkin [144]. The ISGylation of Parkin is catalyzed by the HECT HERC6, which ISGylates two lysine residues of Parkin resulting in the disruption of its auto-inhibitory conformation.

## Modulating HECT E3 activity

In addition to the variety of substrates HECT E3s can target specifically, there is also variety in the type of ubiquitin and ubiquitin-like moieties conjugated. HECT E3substrates may be mono-ubiquitinated or poly-ubiquitinated with additional variation in the lysine linkages in the poly-ubiquitin chains. HECT E3s have been shown to catalyze various poly-ubiquitin chains such as Lys48-linked Ub chains, Lys63-linked Ub chains, or other linkage types [21, 22, 145, 146]. The resulting poly-ubiquitin chains can have different configurations ranging from linear to branched chains that each can consist of homotypic or mixed linkages. In addition to ubiquitin, ubiquitin-like modifiers such as NEDD8 and ISG15 can also be catalyzed by HECT E3 ligases. UBL modifiers can even be part of ubiquitin polymers [147], further emphasizing the wide variety of moieties that can possibly be conjugated by HECT E3s. Depending on the type of moiety, the substrate can be committed to divergent fates or display altered functions. Importantly, various mechanisms exist that can regulate HECT E3 ligases to determine what type of ubiquitin and UBL mono- or polymers they catalyze.

An important determinant in the ubiquitin chain formation is the processivity of the E3 ligase. To efficiently form polyubiquitin chains, some HECT E3s have a processivity site (also called the “exosite”) in the N-lobe that binds the last ubiquitin of the growing chain to keep it in position for catalysis of the next ubiquitin. The processivity site interacts non-covalently with ubiquitin utilizing a series of predominantly hydrophobic residues to contact an interaction surface on ubiquitin that involves the canonical Ile44 hydrophobic patch [112, 148, 149]. Experiments using small molecule inhibitors have demonstrated that the processivity of NEDD4.1 can be regulated by targeting the exosite [150]. These small molecule inhibitors specifically bind to the processivity site, thereby disrupting the formation of polyubiquitin chains. Consequentially, NEDD4.1 changes to a distributive E3 ligase resulting in an increased amount of substrates that are mono-ubiquitinated. Whether the modulation of NEDD4.1 by targeting its site of processivity is also utilized as a regulatory mechanism in the cell remains to be determined. Interestingly, however, a recent screen for ubiquitin variants (UbVs) with increased affinity for the exosite has provided useful insights as to how HECT domain activity can be regulated and modulated [30]. Detailed biochemical analysis and HECT-UbV co-crystallization experiments revealed that UbV binding at the N-lobe exosite could modulate E3 ligase activity through a variety of mechanisms. For example, binding of the UbV to the NEDD4.2 and Rsp5 exosite promoted the transfer of ubiquitin from E2 to E3, whereas other UbV interactions promoted ubiquitin transfer from E3 to the substrate. Another two UbVs were shown to modulate NEDD4.2 activity by decreasing processivity and increasing distributive multi-mono-ubiquitination. As the engineered UbVs are highly specific for one HECT E3 [30] by way of their design and selection process, their specificity may be useful to target and modulate a specific HECT E3 ligase without affecting other HECT E3s in the cell.

To create certain ubiquitin polymers, HECT E3 ligases may also cooperate with other E3 ligases. For example, it was shown that a yeast HECT E3 Ufd4 can team up with the RING domain E3 ligase Ubr1 to poly-ubiquitinate its substrates [151]. The addition of the Ubr1 E3 to the UFD complex resulted in increased processivity towards the Ufd4 substrates. Even though the preference to catalyze different linkage polyubiquitin chains has not been observed in this Ubr1–Ufd4 interaction the cooperation of different E3 ligases may be a good explanation of such ubiquitin polymers.

Phosphorylated ubiquitin has recently been shown to modulate HECT E3 ligase to alter the specific lysine linkage of the polyubiquitin chains it catalyzes. The HECT E3 UBE3C normally forms ubiquitin chains with a mixture of both Lys29 and Lys48 linkages [22]; however, when it uses ubiquitin phosphorylated at serine 20 it preferably poly-ubiquitinates its substrates with Lys48-linked chains [152]. Phosphorylation of ubiquitin on S20 is found, among other PTMs, in mammalian cells [153]; however, the physiological significance of S20 phospho-ubiquitin is currently unknown. The physiological importance of other phospho-ubiquitins such as S65-phosphorylated ubiquitin has been demonstrated. Specifically, the generation of S65 phospho-ubiquitins by PINK1 (PTEN-induced putative kinase 1) was shown to activate the RING-E3 parkin [154]. Thus, it is conceivable that S20-phospho-ubiquitins are also utilized in the cell as a mechanism to regulate E3s and perhaps PTMs of ubiquitin are more commonly involved in the regulation of HECT E3 ligases.

## Summary and significance

A wide variety of mechanisms regulates the substrate specificity and control catalytic activity of the HECT E3 ligases. The domains N-terminal to the HECT domain play a pivotal role in determining substrate specificity, as they are important interfaces that bind to adaptor proteins. Competitive inhibitors or post-translational modifications often control the interactions between adaptors and N-terminal domains, thereby modulating HECT E3 ligase activity.

The intermediate step of transferring ubiquitin from the E2 to the active site of the HECT increases the control exerted by the E3 ligase regarding subsequent catalysis. This contrasts to ubiquitination by RING E3s, where due to the direct transfer of ubiquitin from E2 to the substrate control of the reaction is largely determined by the E2-conjugating enzyme, especially with regards to the linkage type of ubiquitin chain formation. Consequently, mechanisms that control specificity and activity of HECT E3 ligases are of increased importance in determining the fate of HECT E3 substrates. E2-conjugating enzymes remain important regulators of the HECT E3 ligases they cooperate with. The example of the SMURF2–UbcH7 interaction illustrated that the binding affinity of E2 to HECT E3 is an important factor in determining the catalytic activity and processivity of HECT E3s. Blocking of the E2–E3 interaction has also been shown to regulate HECT E3s as seen in the example of ISG15 binding to the E2-binding site of NEDD4.1.

A recurring type of mechanism that controls HECT E3 ligases, especially that of the NEDD4 subfamily, is the self-control of inter- and intramolecular interactions. Through interactions of the HECT domain with N-terminal C2 or WW domains, NEDD4 family members can take up auto-inhibitory folds or form homodimers that block catalytic activity until an external regulator unlocks the HECT E3. Mechanisms that can relieve these auto-inhibitory and oligomeric forms such as post-translational modifications, allosteric protein–protein interactions, and Ca^2+^ interactions have been highlighted for the NEDD4 family in this review. The trimers formed by Rsp5 and E6AP were shown to have opposing regulatory outcomes. Oligomerization using a similar trimerization interface appears to lead to inhibition of Rsp5 and activation of E6AP. To understand how HECT domain-mediated trimerization can lead to these opposing outcomes in activity, it will be key to determine the structural and functional contributions of the region N-terminal to HECT domain.

HECT E3s are capable of catalyzing a variety of ubiquitin polymers and can additionally catalyze the attachment of the ubiquitin-like modifiers such as ISG15 and NEDD8. The processivity of HECT E3 ligases may also be an important factor in determining the outcome for its substrates. Various studies discussed in this review presented regulatory mechanisms that influenced HECT E3 ligase processivity. The lysine linkage of ubiquitin chains is often determined primarily by the HECT E3; however, regulators can modulate HECT E3s to alter the lysine linkage they catalyze. Thus, substrates of HECT E3s can be post-translationally modified with a wide variety of ubiquitin polymers that may have various functional outcomes. For a full understanding of the functional consequences of HECT E3substrate ubiquitination, we need to not only identify these substrates, but also characterize the inter-ubiquitin connectivity of their ubiquitin polymers. While challenging, such an approach has become feasible due to the recent advances in targeted proteomics techniques [155].

Regulation of HECT E3 ligases plays an important part in the determination of cell fates and as such disruption of HECT E3 function is often implicated in disease. Understanding the mechanisms that regulate HECT E3s will be critical in the search for treatments that intervene with or enhance HECT E3 functions. Various diseases have been associated with HECT E3 function. For example, increased E6AP activity has been associated with autism and most cervical cancers [56, 126], whereas reduced activity of E6AP has been implicated in Angelman syndrome [9, 10]. Regulating HECT E3s by targeting their upstream regulators may be a promising strategy. For example, a study showed that stimulating PKA by using pharmacological agents was an effective method to turn down E6AP activity in neurons [126]. While most strategies using pharmacological agents may focus on inhibition, in some cases activation or modulation of the HECT E3 might be a more appropriate strategy. The screen using ubiquitin variants (UbV) found various sites on the HECT domain that upon interaction with an UbV resulted in activation or modulation of the HECT E3. Perhaps pharmacological agents can be developed that target the same activation or modulation sites as a therapeutic strategy. The wide variety of regulatory mechanisms that control HECT E3s in the cell should serve as an indicator of the many possible avenues that can be taken in order to manipulate the activity of HECT E3 ligases.
